# High Burden of Non-Influenza Viruses in Influenza-Like Illness in the Early Weeks of H1N1v Epidemic in France

**DOI:** 10.1371/journal.pone.0023514

**Published:** 2011-08-17

**Authors:** Nathalie Schnepf, Matthieu Resche-Rigon, Antoine Chaillon, Anne Scemla, Guillaume Gras, Oren Semoun, Pierre Taboulet, Jean-Michel Molina, François Simon, Alain Goudeau, Jérôme LeGoff

**Affiliations:** 1 Université Paris Diderot, Sorbonne Paris Cité, Microbiology Department, Saint-Louis Hospital, APHP, Paris, France; 2 François Rabelais University, Microbiology Department, Bretonneau Hospital, CHRU Tours, France; 3 Université Paris Diderot, Sorbonne Paris Cité, Biostatistics Department, Saint-Louis Hospital, APHP, Paris, France; 4 Université Paris Diderot, Sorbonne Paris Cité, Infectious Diseases Department, Saint-Louis Hospital, APHP, Paris, France; 5 Internal Medicine and Infectious Diseases Departement, Bretonneau Hospital, CHRU Tours, France; 6 Emergency Department, Saint-Louis Hospital, APHP, Paris, France; 7 Inserm U941, Institut Universitaire d'Hématologie, Paris, France; Naval Research Laboratory, United States of America

## Abstract

**Background:**

Influenza-like illness (ILI) may be caused by a variety of pathogens. Clinical observations are of little help to recognise myxovirus infection and implement appropriate prevention measures. The limited use of molecular tools underestimates the role of other common pathogens.

**Objectives:**

During the early weeks of the 2009–2010 flu pandemic, a clinical and virological survey was conducted in adult and paediatric patients with ILI referred to two French University hospitals in Paris and Tours. Aims were to investigate the different pathogens involved in ILI and describe the associated symptoms.

**Methods:**

H1N1v pandemic influenza diagnosis was performed with real time RT-PCR assay. Other viral aetiologies were investigated by the molecular multiplex assay RespiFinder19®. Clinical data were collected prospectively by physicians using a standard questionnaire.

**Results:**

From week 35 to 44, endonasal swabs were collected in 413 patients. Overall, 68 samples (16.5%) were positive for H1N1v. In 13 of them, other respiratory pathogens were also detected. Among H1N1v negative samples, 213 (61.9%) were positive for various respiratory agents, 190 in single infections and 23 in mixed infections. The most prevalent viruses in H1N1v negative single infections were rhinovirus (62.6%), followed by parainfluenza viruses (24.2%) and adenovirus (5.3%). 70.6% of H1N1v cases were identified in patients under 40 years and none after 65 years. There was no difference between clinical symptoms observed in patients infected with H1N1v or with other pathogens.

**Conclusion:**

Our results highlight the high frequency of non-influenza viruses involved in ILI during the pre-epidemic period of a flu alert and the lack of specific clinical signs associated with influenza infections. Rapid diagnostic screening of a large panel of respiratory pathogens may be critical to define and survey the epidemic situation and to provide critical information for patient management.

## Introduction

In order to monitor the spread of influenza and alert health handlers, several epidemiological tools have been developed. In France, a network of 1300 general practitioners, “Réseau Sentinelles”, working throughout the country, provides real-time clinical data used to evaluate regional and national influenza spreading [1], [2]. The criteria used by this network to define clinical influenza-like illness (ILI) are the occurrence of a sudden fever above 39°C with myalgia and respiratory signs. In general no formal viral diagnosis is carried out. The Groupes Régionaux d'Observation de la Grippe (GROG) is a second French network that surveys the emergence and the spread of the influenza viruses [3], [4]. This network is based on clinical surveillance of acute respiratory infections and laboratory analysis of nasal specimens collected from adults and children by volunteer general practitioners and pediatricians.

According to the sentinel network's criteria, French health authorities proclaimed that flu epidemic level was reached during the second week of September 2009 (week 37) [5], [6]. On the contrary, data provided by the GROG showed only sporadic H1N1v activity until the last week of October (week 44) [6], [7]. Thus, it became rapidly obvious that a variety of viruses were circulating in the community and that an overestimation of myxovirus infection was at stake [8], [9], [10], [11].

As a better knowledge of the epidemic status was a key feature for national healthcare organization, hospital preparedness, patient management and disease control, unambiguous viral diagnosis appeared critical. In France, data on viral aetiologies associated with ILI were at best sporadic and correlations with clinical symptoms were often lacking. Extensive molecular assays to screening for respiratory viruses were not available countrywide for routine diagnosis. Therefore the epidemiological pattern of respiratory pathogens with overlapping seasonality was poorly known.

The aim of the present study was to investigate respiratory pathogens involved in ILI during the early weeks of the 2009–2010 H1N1v diffusion in France (weeks 35 through 44) and describe the associated symptoms in paediatric and adult populations.

## Materials and Methods

### Ethics Statement

This study was a non-interventional study with no addition to usual proceedures. Biological material and clinical data were obtained only for standard viral diagnostic following physicians' prescriptions (no specific sampling, no modification of the sampling protocol, no supplementary question in the national standardized questionnaire). Data analyses were carried out using an anonymized database. According to the French Health Public Law (CSP Art L 1121-1.1), such protocol does not require approval of an ethics committee and is exempted from informed consent application.

### Patients and samples

In the two academic hospitals, Saint-Louis hospital (SLS) in Paris and Tours hospital (TRS), influenza-like illness (ILI) was defined as a patient suffering from at least one general symptom (fever above 38°C, asthenia, myalgia, shivers or headache) and one respiratory symptom (cough, dyspnoea, rhinitis or pharyngitis), in agreement with the guidelines from the French Institut de Veille Sanitaire (InVS), a governmental institution responsible for surveillance and alert in all domains of public health [12]. Criteria for severe clinical presentation were temperature below 35°C or above 39°C despite antipyretic, cardiac frequency above 120/min, respiratory frequency above 30/min, respiratory distress, systolic arterial pressure below 90 mmHg or altered consciousness. Predisposing factors of critical illness were children younger than one year old, pregnant women, diabetes, chronic pre-existing disease (such as respiratory, cardiovascular, neurologic, renal, hepatic or hematologic diseases) and immunosuppression (associated with HIV infection, organ or hematopoietic stem cells transplantation, receipt of chemotherapy or corticosteroids) [13], [14]. A cluster of suspected influenza infections was defined as at least three possible cases in a week in a closed community (household, school,…) [15].

In the two institutions, the prescription of H1N1v molecular testing was recommended for patients with ILI and with either a severe clinical presentation, an underlying risk factor of complications or a condition which was not improving under antiviral treatment. Investigation of grouped suspected cases was also recommended. From week 35 (last week of August) to 44 (last week of October), 413 endonasal swabs were collected in 3 ml of Universal Transport Medium (Copan Diagnostics Inc, Murrieta, CA) from adults and children seen in emergency rooms for suspected ILI (Table 1) and sent to SLS and TRS laboratories for H1N1v detection. The two microbiology laboratories participated in the reference laboratories network for the detection of pandemic influenza H1N1v.

**Table 1:** Number of endonasal swabs sent to SLS and TRS laboratories for H1N1v detection during weeks 35 through 44.

Week number (2009)	Global	SLS	TRS
**35**	2	2	0
**36**	2	2	0
**37**	35	5	30
**38**	69	12	57
**39**	64	17	47
**40**	42	13	29
**41**	29	9	20
**42**	28	12	16
**43**	48	27	21
**44**	94	44	50
***Total***	***413***	***143***	***270***

Clinical data were collected at the time of medical attention and reported by clinicians on a national standardized questionnaire provided by InVS [1], [12]. This questionnaire included the presence or absence of the main general and respiratory symptoms associated with ILI (fever, asthenia, myalgia, shivers, headache, cough, rhinitis, pharyngitis, sudden onset) [12].

### Detection of H1N1v pandemic influenza A virus and other respiratory viruses

Total nucleic acid was extracted from 400 µL of Universal Transport Medium using the EasyMag System (Biomérieux, Marcy l'Etoile, France) in SLS or the EZ1 Advanced XL (Qiagen, Courtaboeuf, France) in TRS, according to the manufacturers' instructions (elution volume: 100 µL in SLS or 90 µL in TRS). Before extraction, 5 µl of an Internal Amplification Control (IAC) which contained an encephalomyocarditis virus (EMC) RNA transcript was added into the sample.

Pandemic H1N1v infection was diagnosed by real-time reverse transcription–PCR (RT-PCR) assay on a 7500 Real Time PCR System (Applied Biosystems, Foster City, CA) according to the protocol of the Centers for Disease Control (CDC) [16]. Other respiratory infections were investigated by a multiplex molecular assay based on the Multiplex Ligation-dependent Probe-Amplification (MLPA) technology (RespiFinder19®, Pathofinder, Maastricht, The Netherlands) that allows the detection and differentiation of 14 respiratory viruses, including influenza virus A (InfA), influenza virus B (InfB), rhinovirus (RHV), parainfluenza viruses 1 to 4 (PIV-1 to PIV-4), human metapneumovirus (hMPV), adenovirus (ADV), respiratory syncytial virus A (RSVA), respiratory syncytial virus B (RSVB) and human coronaviruses 229E, OC43 and NL63 (Cor-229E, Cor-OC43, Cor-NL63) [17]. The test allows also the detection of H5N1 influenza A virus and of four bacteria: *Chlamydophila pneumoniae* (CP), *Mycoplasma pneumoniae* (MP), *Legionella pneumophila* (LP) and *Bordetella pertussis* (BP). The amplified MLPA products were analyzed on an ABI 3100 genetic analyzer (Applied Biosystems, Foster City, CA). Fragment sizing analysis was performed with the GeneMarker software (SoftGenetics, LLC, State College, PA).

Further testing for H1N1v was carried out with Simplexa™ Influenza A H1N1 (2009) (Focus Diagnostics, Cypress, California) when the CDC real time RT-PCR assay was negative for H1N1 and the RespiFinder19® assay was positive for Influenza A. If this latter assay was negative, H3N2 typing was performed as previously described [18].

### Statistical analysis

Data from our study are summarized as frequencies and percentages for categorical variables. Quantitative variables are presented as medians, 25th and 75th percentiles. To compare those variables according to the viral infection status, Fisher tests were performed for categorical variables and Wilcoxon tests for quantitative variables. To assess the increase of infectious proportion above the weeks, a Chi square trend test was used. All tests were two-sided at the 0.05 significance level. Analyses were performed using R.2.10.1 statistical package (R Development Core Team, R: A Language and Environment for Statistical Computing, 2009, Vienna, Austria, http://www.R-project.org). We choose to not plot the percentage of positive samples for week 35 and week 36. Indeed the small number of tested patients during those two weeks doesn't allow us to obtain precise enough estimations of the prevalence of endonasal swabs positive for H1N1v or for other respiratory pathogens.

## Results

### H1N1v detection during weeks 35 through 44

**Figure 1:**
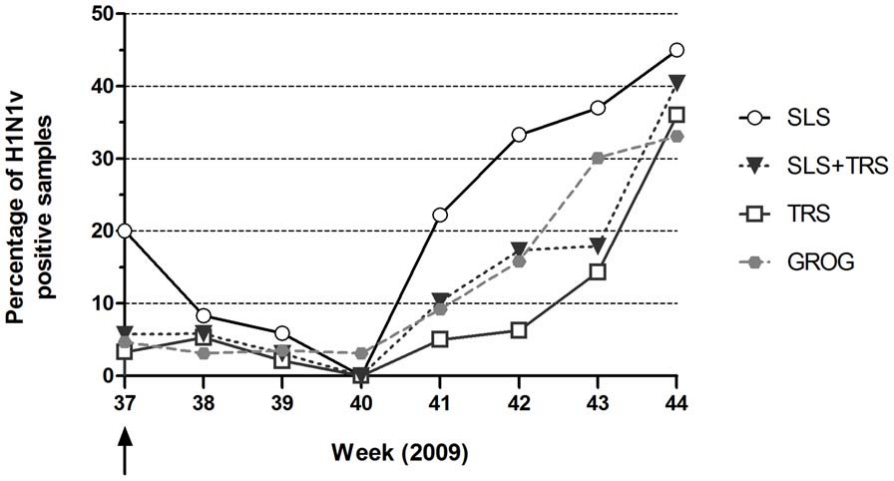
Weekly rate of endonasal swabs positive for H1N1v pandemic influenza virus. Percentage of H1N1v positive endonasal swabs are indicated for each hospital (SLS: open circle, TRS: open square) and for both (plain black triangle and black dotted line). The national weekly rate from data provided by the GROG network is indicated by a grey dotted line and plain grey circles. The epidemic status of H1N1v was proclaimed in France by health authorities during the second week of September (black arrow).

By using CDC reference assay, H1N1v was detected in 66 samples out of 413 (16.6%), more frequently in SLS (38 samples) than in TRS (28 samples) (p<10^−4^). Overall, weekly percentage of H1N1v positive endonasal swabs remained under 10% until week 41 and increase significantly after (P_Trend_<0.0001) (Figure 1). Rate of H1N1v detection reached 30% in SLS at week 42 and in TRS at week 44. Overall, this rate was in agreement with results provided by the GROG network, showing an earlier start of H1N1v epidemic in Paris area [7], [19].

### Detection of other respiratory pathogens during weeks 35 through 44

**Figure 2:**
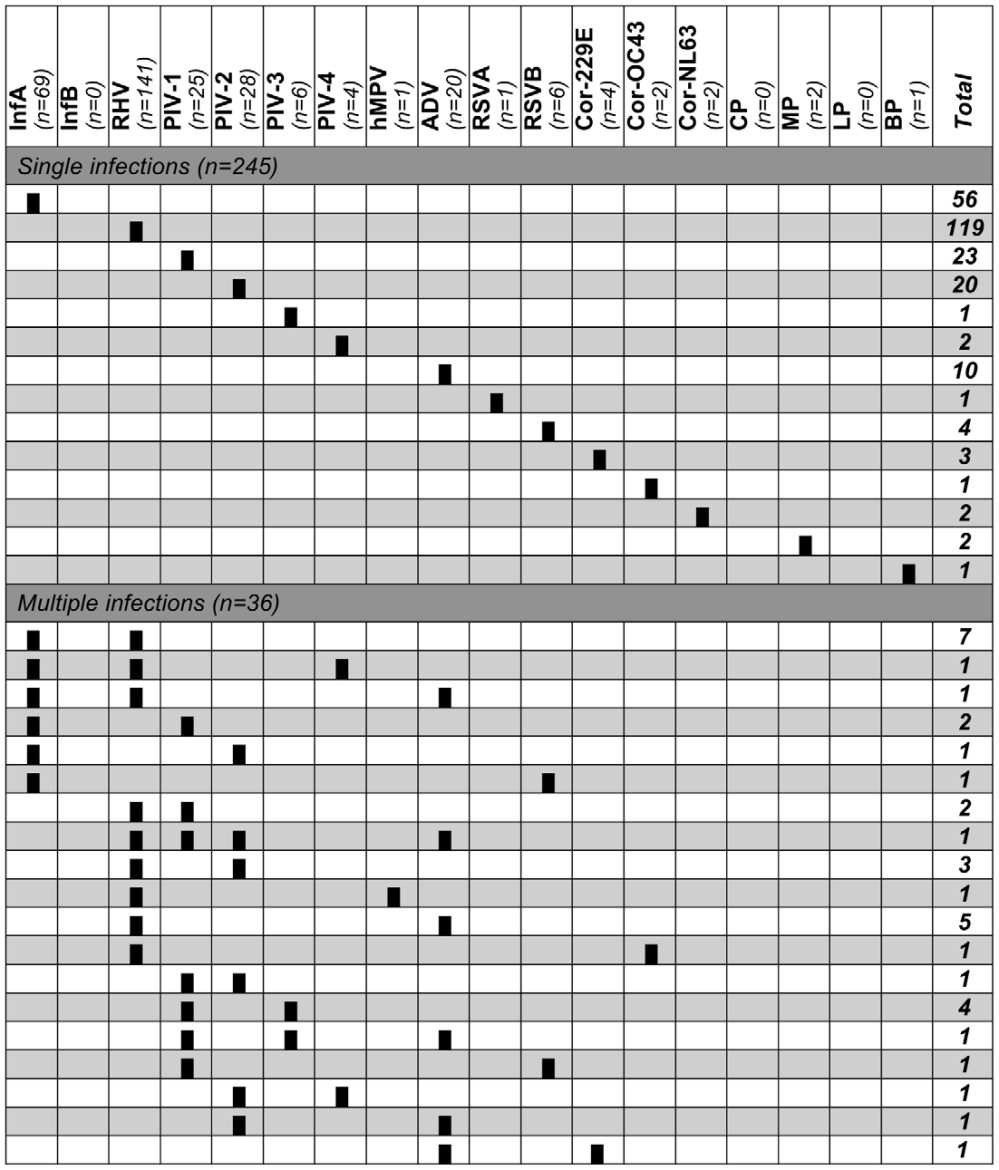
Aetiologies of influenza-like illness. For each pathogen, the number of patients in whom this pathogen was detected (including single and multiple infections) is indicated in italic at top. The different patterns of single and multiple infections (1 line = 1 pattern) are depicted by the presence of plain black rectangles for relevant pathogens. The total of samples for each pattern is indicated in bold at the end of the line. InfA: influenza virus A; InfB: influenza virus B; RHV: rhinovirus; PIV-1 to PIV-4: parainfluenza virus 1 to 4; hMPV: human metapneumovirus; ADV: adenovirus; RSVA and B: respiratory syncytial virus A and B; Cor-229E, Cor-OC43, Cor-NL63: human coronaviruses 229E, OC43 and NL63; CP: *Chlamydophila pneumoniae*; MP: *Mycoplasma pneumoniae*; LP: *Legionella pneumophila*; BP: *Bordetella pertussis*.

All 413 nucleic acid extracts were analyzed using the RespiFinder19® assay (Figure 2). Sixty six patients tested H1N1v positive with CDC real time RT-PCR assay were confirmed with the multiplex assay. Thirteen were also co-infected by one or two other respiratory pathogens (multiple infections) (Figure 2). Three of the 347 H1N1v negative samples could not be studied with the multiplex assay because they contained RT-PCR inhibitors (no amplification of the internal control). Two hundred and fifteen (62.5%) of the remaining 344 H1N1v negative samples were found positive for at least one respiratory pathogen (Figure 2). Two hundred and twelve were positive for non influenza pathogens (189 single infections and 23 mixed infections with two, three or four viruses) and three additional single infections by influenza A were identified in SLS, including two by pandemic H1N1v and one by seasonal H3N2, as determined after molecular typing (data not shown).

Overall, 68 patients (16.5%) were then positive for H1N1v, one for H3N2 and 212 for non influenza pathogens. There were 245 single infections (55 with H1N1v and 190 with other respiratory pathogens) and 36 mixed infections (13 with H1N1v and 23 without H1N1v) (Figure 2).

Among H1N1v negative single infections, the most prevalent viruses were rhinovirus (62.6%, 119 patients), followed by parainfluenza viruses 1 to 4 (24.2%, 46 patients), adenovirus (5.3%, 10 patients), human coronavirus 229E, OC43 and NL63 (3.2%, 6 patients) and respiratory syncytial virus A and B (2.6%, 5 patients) (Figure 2). In addition, RespiFinder19® assay identified three patients with bacterial infection, two with *Mycoplasma pneumoniae* (one 25 years old female in SLS and one 39 years old female in TRS) and one with *Bordetella pertussis* (one 60 years old male in SLS). No single infection by influenza B, hMPV, *Chlamydophila pneumoniae* or *Legionella pneumophila* was identified (Figure 2).

In mixed infections, PIV (1 to 4) and RHV were the most frequent (75% [27/36] and 61.1% [22/36], respectively), followed by H1N1v (36.1% [13/36]), ADV (27.8% [10/36]) and RSV-B (5.6% [2/36]) (Figure 2). Co-detection or multi-detection were very frequent along with adenovirus infection (50% [10/20]), PIV infection (37.0% [27/73]) including mixed infections with several types and less frequently with rhinovirus infection (15.6% [22/141]). The frequency of viral co-infection was slightly higher in samples positive for H1N1v as compared to samples positive for other respiratory pathogens, but without significance (19.1% [13/68] *vs.* 10.8% [23/213]). RHV was, for instance, the more frequent co-pathogen in H1N1v positive patients (13.2% [9/68]). To analyze if viral co-infections occurred more frequently for some viruses, we carried out a two by two comparisons, that showed a higher proportion of co-infection only for ADV (p = 0.05).

**Figure 3:**
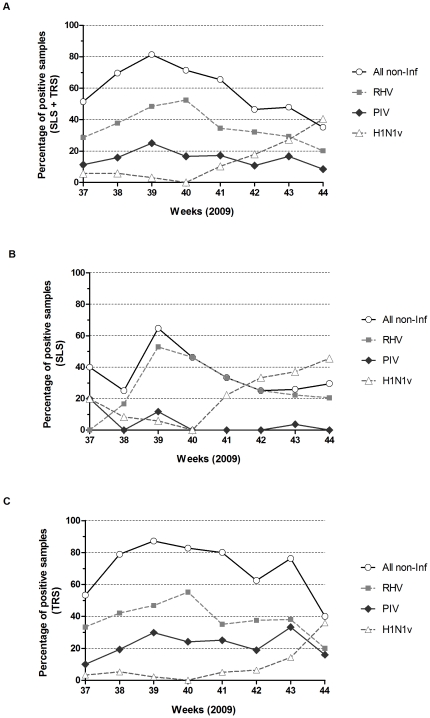
Weekly detection of H1N1v *versus* non-influenza respiratory viruses in endonasal swabs. Frequencies of weekly detection are represented in the overall studied population (A), in samples from Saint-Louis hospital (B) or Tours hospital (C), with open circles for non-influenza respiratory viruses (all non-Inf), plain squares for rhinoviruses (RHV), plain diamonds for parainfluenza viruses (PIV) and open triangles for H1N1v. All viruses involved in the co-infections were counted individually.

Non-influenza respiratory viruses presented a different epidemic profile compared to H1N1v. Overall, in both hospitals, weekly rate of non-H1N1v respiratory viruses whether alone or involved in co-infection increased between week 37 and 39 (from 51.4% to 81.3%) and then consistently decreased (Figure 3). RHV infections that represented nearly half of non-H1N1v viral infections (141 out of 213, 66.2%) were a significant contributing factor. In both hospitals, emergence of H1N1v cases was associated with a rapid decline of RHV rate of infection from 50–60% down to less than 20% with a one to two weeks gap between SLS and TRS.

### Clinical characteristics

Data on age (Table 2) and gender were available for all 413 patients seen for ILI in both hospitals.

**Table 2:** Age of patients with respiratory samples positive for H1N1v, positive for other respiratory pathogens or negative.

		Global	H1N1v positive	H1N1v negative	
				Other respiratory pathogens	No respiratory pathogens*
		Number (%)(n = 143)	Number (%)(n = 68)	Number (%)(n = 213)	Number (%)(n = 132)
**Age (in years)**	<15	124 **(30.0)**	16 **(23.5)**	90 **(42.3)**	18 **(13.6)**
	15–39	157 **(38.0)**	32 **(47.1)**	68 **(31.9)**	57 (**43.2)**
	40–64	103 **(25.0)**	20 **(29.4)**	34 **(16.0)**	49 **(37.1)**
	≥ 65	29 **(7.0)**	0 **(0.0)**	21 **(9.8)**	8 **(6.1)**

*These data include the 3 patients whose respiratory samples could not be studied with the multiplex assay because of RT-PCR inhibitors.

Overall, 124 of the 413 patients (30.0%) were less than 15 years old (4 in SLS and 120 in TRS) and 281 patients (68.0%) were under 40 years of age (68 in SLS and 213 in TRS). In SLS, the median population age was 41 (Interquartile range [IQR]: 28–56) with 49.7% being males, whereas in TRS, the median population age was 17 ([IQR = 3–34]) with 51.1% being males.

**Figure 4:**
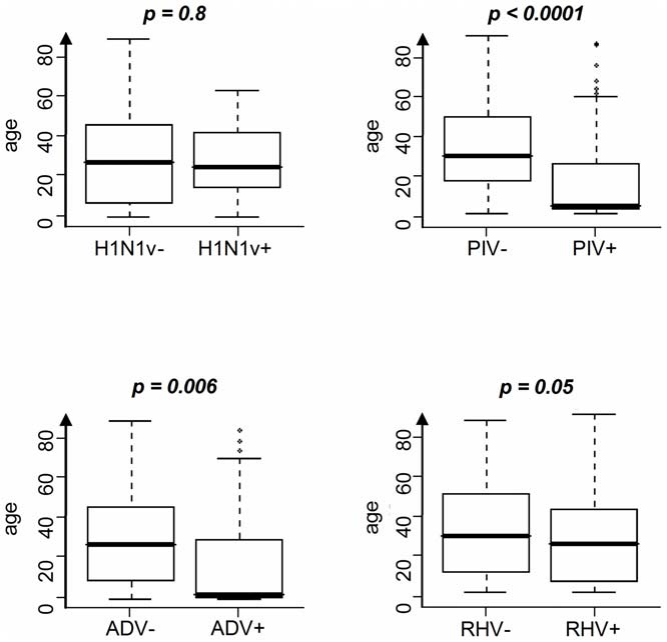
Association between age and respiratory viral infections. For each distribution, the horizontal lines represent the 10th, 25th, 50th (median), 75th, and 90th percentiles. Comparison used Wilcoxon's test. H1N1v: H1N1v pandemic influenza virus; PIV: parainfluenza virus; ADV: adenovirus; RHV: rhinovirus.

In both institutions, 85.5% (106/124) children younger than 15 years of age were infected by at least one respiratory pathogen (Table 2). H1N1v infected patients were not significantly younger than H1N1v non infected patients (27 years old *vs*. 25 years old, p = 0.80) (Figure 4). However, 70.6% (48/68) of H1N1v cases were identified in patients under 40 years old (22 in SLS and 26 in TRS) and no case was observed in patients older than 65 years (Table 2). PIV infection occurred in very young patients (median age = 4 *vs.* 29 for patients without PIV, p<10^−4^) (Figure 4). The same observation was made for ADV infection (median age = 2.5 *vs.* 25 for patients without ADV, p = 0.006) (Figure 4). Consequently, PIV and ADV were more frequently detected in the younger population of TRS *versus* SLS (p<10^−4^ and p<10^−3^ respectively). In contrast, although individuals with RHV infection were slightly younger than individuals without (median age = 24 *vs.* 29 for patients without RHV, p = 0.05) (Figure 4), influenza-like illness associated with RHV was more frequent in SLS than in TRS (p = 0.012). Finally, patients with viral multiple infection were significantly younger than those with single infection (median, IDR: 4, 2–18.5 *vs.* 25, 6–43) and rates of mixed infection were significantly higher in patients under 15 years as compared to older ones (19.4% *vs.* 4.1%, p<0.0001).

At the time of medical attention, 383 (92.7%) standardized clinical questionnaires were collected out of 413 patients. Four of them could not be exploited because they were too incomplete. A review of the 379 workable questionnaires showed that 90.8% (344/379) of the patients included in this study fulfilled the criteria of ILI as defined above, and 52.5% had either a severe clinical presentation or an underlying risk factor of complications (45.9%, 174/379), or were in a suspected cluster of grouped cases (6.6%, 25/379).

Overall, most patients have fever (93.9%) and cough (86.1%) (Table 3). Other classical clinical signs associated with ILI such as asthenia, myalgia, shivers, headache, rhinitis or pharyngitis were less frequent. A sudden onset was also described in 59.2% of cases. Only 32.5% of the patients had a temperature above 39°C; the age of these patients ranged from zero to 86 years, with a median age of 32 years and a mean age of 34 years (data not shown).

**Table 3:** Clinical characteristics of patients with respiratory samples positive for H1N1v, positive for other respiratory pathogens or negative.

		Global	H1N1v positive	H1N1v negative		*p* ∥ ††	*p* ^¶^ ††	*p* ^**^ ††
				Other respiratory pathogens	No respiratory pathogens§			
		Number (%) (n = 413)	Number (%) (n = 68)	Number (%) (n = 213)	Number (%) (n = 132)			
**Fever**	w/*	354 **(93.9)**	56 **(98.2)**	177 **(90.8)**	121 **(96.8)**	**0.036**	0.08	1
	w/o†	23 **(6.1)**	1 **(1.8)**	18 **(9.2)**	4 **(3.2)**			
	N/A‡	26	11	18	7			
**Asthenia**	w/	140 **(57.6)**	25 **(50.0)**	60 **(57.1)**	55 **(62.5)**	0.37		
	w/o	103 **(42.4)**	25 **(50.0)**	45 **(42.9)**	33 **(37.5)**			
	NA	160	18	108	44			
**Myalgia**	w/	195 **(58.4)**	28 **(52.8)**	98 **(59.0)**	69 **(60.0)**	0.68		
	w/o	139 **(41.6)**	25 **(47.2)**	68 **(41.0)**	46 **(40.0)**			
	N/A	69	15	47	17			
**Shivers**	w/	72 **(32.0)**	16 **(31.4)**	28 **(30.8)**	28 **(33.7)**	0.93		
	w/o	153 **(68.0)**	35 **(68.6)**	63 **(69.2)**	55 **(66.3)**			
	N/A	178	17	122	49			
**Headache**	w/	95 **(40.8)**	17 **(33.3)**	40 **(42.1)**	38 **(43.7)**	0.48		
	w/o	138 **(59.3)**	34 **(66.7)**	55 **(57.9)**	49 **(56.3)**			
	N/A	170	17	118	45			
**Cough**	w/	315 **(86.1)**	51 **(89.5)**	173 **(90.1)**	91 **(77.8)**	**0.01**	1	0.09
	w/o	51 **(16.9)**	6 **(10.5)**	19 **(9.9)**	26 **(22.2)**			
	N/A	37	11	21	15			
**Rhinitis**	w/	103 **(42.9)**	19 **(37.3)**	53 **(52.0)**	31 **(35.6)**	0.053		
	w/o	137 **(57.1)**	32 **(62.7)**	49 **(48.0)**	56 **(64.4)**			
	NA	163	17	111	45			
**Pharyngitis**	w	54 **(24.1)**	5 **(10.0)**	31 **(34.4)**	18 **(21.4)**	**0.003**	**0.001**	0.10
	w/o	170 **(75.9)**	45 **(90.0)**	59 **(65.6)**	66 **(78.6)**			
	N/A	179	18	123	48			
**Sudden onset**	w/	141 **(59.2)**	27 **(54.0)**	58 **(57.4)**	56 **(64.4)**	0.42		
	w/o	97 **(40.8)**	23 **(46.0)**	43 **(42.6)**	31 **(35.6)**			
	N/A	165	18	112	45			

*w/: presence of the clinical sign,

† w/o: absence of the clinical sign,

‡ N/A: not available.

§ These data include the three patients whose respiratory samples could not be studied with the multiplex assay because of RT-PCR inhibitors.

∥ Comparison between the three groups of patients (with respiratory samples respectively positive for H1N1v, positive for other respiratory pathogens or negative). If p value is significant (<0.05), patients positive for H1N1v are compared with patients positive for other respiratory pathogens ^(¶)^ and with patients without any detection of respiratory pathogens ^(**)^.

†† Significant p values (<0.05) are indicated in bold.

In H1N1v infected patients (including single and multiple infections), the main symptoms were also fever (98.2%) and cough (89.5%) (Table 3). Similar median temperature was reported in H1N1v positive and in H1N1v negative patients (39 [IQR = 35.5–41] *vs.* 38.8 [IQR = 37.8–40.4], p = 0.68). The proportion of patients with a temperature above 39°C was not different (H1N1v positive: 34.3% *vs.* H1N1v negative: 32.3%, p = 0.84) (data not shown).

We then compared clinical characteristics between patients positive for H1N1v, patients positive for other respiratory pathogens and negative for H1N1v and patients without any detection of respiratory pathogens (as detected with RespiFinder19®) (Table 3). There was no difference between the three groups except for fever, cough, pharyngitis. However for these latter symptoms, the comparison between patients positive for H1N1v and those positive for other respiratory pathogens or between patients positive for H1N1v and those without any detection of respiratory pathogens, showed no difference except for pharyngitis, which was less frequent in patients positive for H1N1v than in patients positive for other respiratory pathogens (Table 3).

As RHV was the most frequent aetiology in ILI, we also compared clinical symptoms observed in patients with a single infection by RHV or by H1N1v (data not shown). There was no difference except that rhinitis and pharyngitis were significantly more frequent in RHV infection (62.7% *vs.* 34.1% [p = 0.006] and 39.0% *vs.* 10.0% [p = 0.001], respectively).

Viral multiple infection (including samples with H1N1v) was not associated with a different clinical presentation. Fever and cough were observed in over 90% of the patients (90.6% and 90.3%, respectively), but only 33.3% of these patients had a temperature above 39°C, which was not different from patients with single viral infection (28.6%).

## Discussion

Our results highlight the high frequency of non-influenza viruses involved in acute respiratory infections during the epidemic period of a flu alert as defined by the Réseau Sentinelles according to ILI definition (a sudden fever above 39°C accompanied by myalgia and respiratory signs). These data extent previous observations in Europe reporting high prevalence of RHV infections before seasonal influenza [4], [20] or in 2009, before H1N1v pandemic influenza [1], [8], [9], [11], [21]. We confirm that RHV represent the most frequent aetiology of acute respiratory infections both in adult and paediatric populations and may represent more than 50% of cases. We show that other viral infections than influenza and RHV may represent up to 30% of aetiologies. We observed differences between the two hospitals, with a higher frequency of parainfluenza and ADV infections in Tours in contrast with a higher frequency of RHV in Paris, likely explained by the higher proportion of paediatric samples collected in Tours. However, despite the distance between the two institutions (about 250 km) and differences between the two populations, both presented similar patterns of high frequency of non-influenza viruses in acute respiratory infections before the flu epidemic wave and a decline when influenza reached epidemic levels.

In the two cities, high frequencies of RHV were seen at the same level with a likely different evolution speed, with sudden increase and decrease in SLS and more progressive variation in TRS. In both institutions, there was a decrease in the proportion and number of RHV diagnoses roughly in parallel with the increase of influenza diagnoses. Indeed, H1N1v exceeds 20% of positive detection's rate only when RHV dropped under 40%. These data are thus consistent with negative interaction of the two epidemics at the population level. It was previously hypothesised that RHV epidemic could interfere with the spread of pandemic influenza [20], [21], [22]. Few *in vitro* data support this hypothesis. It has been reported that interferon and other cytokines production by RHV infected cells induced a refractory state to virus infection of neighbouring cells [23]. Further work is needed to confirm *in vitro* and *in vivo* such negative interactions and if viral interference are really translated to a population level. Analysis of rhinovirus and influenza epidemics in previous years should also help to determine if similar interferences were observed with seasonal influenza and to elaborate modelling and prediction of the spread of influenza according to respiratory viruses' circulation. Systematic extensive screening of respiratory viruses at a national level should be implemented for this purpose.

Very few RSV infections were observed in contrast to usual epidemiology which was characterized the last four past years by a start of epidemics in weeks 44–45 [1]. It has been confirmed by other laboratories and the French InVS that the 2009–10 RSV epidemic was delayed and had a lower impact compared with the previous winter season [1], [24]. Delayed and reduced RSV spread may be due to viral interference between RSV and influenza. Another possible explanation is better prevention behaviour about respiratory infections as recommended by a national campaign including recommendations for hands washing after sneezing and the use of mask [1].

Influenza infections were mainly detected in patient under 40 years old and no case was found in patients older than 65. These results corroborate previous data suggesting that past seasonal H1N1 infections or vaccination may give partial crossed protection [10], [13], [25]. We have previously shown that the neutralizing titers against pandemic H1N1v virus correlate significantly with neutralizing titers against a seasonal H1N1 virus, and that the H1N1v pandemic influenza virus neutralizing titer was significantly higher in subjects who had recently been inoculated by a seasonal trivalent influenza vaccine [26].

Viral co-infections were predominantly seen in paediatric patients, as previously described [4], [27], [28], [29], both in influenza and non-influenza cases at a similar rate. No evidence of more pronounced respiratory impact was seen in these patients.

Our results showed the lack of specific clinical signs associated with proven H1N1v infections. Clinical characteristics did not differ between influenza infections or other viral infections. In particular, the proportion of patients with fever above 39°C was not higher in H1N1v positive patients. In addition, the patients without any evidence of respiratory viral infections did not have different symptoms. These patients may have been infected with other virus not included in the multiplex assay (human Bocavirus, coronavirus HKU1) [9], [10], [11] or were seen too late at the time of viral shedding was cleared [30]. However, to determine how specific the symptoms are for influenza would require to assess also the distribution of respiratory pathogens (H1N1v and other respiratory viruses) and related symptoms in patients presented at the emergency departments in SLS and TRS with respiratory syndromes, but not tested for H1N1v. In addition, despite some underlying conditions that were associated with complications not previously observed in seasonal influenza, most illnesses caused by the H1N1v virus were acute and self-limited [13], [31]. The higher proportion of non influenza viruses reported in ILI in 2009 was thus most likely a consequence of more frequent visits to a doctor for respiratory tract infections than usually observed for fear of the flu pandemic. The general lack of difference in symptoms in the particular context of H1N1v pandemic has therefore to be considered with caution and does not rule out that more significant differences may arise in future influenza epidemics with other influenza viruses. Our data confirm that it may be virtually impossible to recognize symptoms heralding H1N1v infections and virological data should be helpful along with clinical reports to monitor influenza epidemic [10].

Molecular multiplex detection has recently emerged as a potent diagnostic tool to determine acute respiratory infections' aetiologies [11], [32], [33]. These data show that sensitive molecular multiplex detection of respiratory viruses is feasible and efficient for the detection of virus involved in acute respiratory infections and provides insights into their epidemic profile. Our results confirm the performance of RespiFinder19® assay to detecting respiratory viruses in the general population as recently shown in transplant patients with ILI [34]. RespiFinder19® confirmed all H1N1 infections detected by the CDC reference assay and was able to identify two additional H1N1 cases suggesting a high sensitivity of this multiplex assay to detect influenza A infections.

In conclusion, our results highlight that successive and mixed outbreaks of respiratory viral infections may affect influenza epidemiology and can lead to misinterpret the early development of a flu epidemic. Rapid diagnostic screening of a large panel of respiratory pathogens may be critical to define and survey the epidemic situation and to provide critical information for patient management.

## Acknowledgments

We gratefully acknowledge the contribution of the members of the two virology laboratories and Sandrine Picco for their excellent technical assistance in the detection of H1N1v pandemic virus and other respiratory viruses, and Catherine Scieux for her help in epidemiological data analysis.
